# Artificial Light at Night Alters Both of Nocturnal and Diurnal Pollination Networks in Alpine Meadows

**DOI:** 10.1002/ece3.74368

**Published:** 2026-10-07

**Authors:** Ningna Lu, Yinyin Wang, Wei Cai, Yangyang Chen, Lifang Yin, Xiuke Chen, Jie Cao, Qianqian Luo

**Affiliations:** ^1^ College of Life Science Northwest Normal University Lanzhou China

**Keywords:** alpine meadows, artificial light at night, cascading effects, pollination networks

## Abstract

Artificial light at night (ALAN) is an increasingly pervasive driver of global change that disrupts natural diel rhythms and alters species interactions. However, its effects on plant–pollinator networks across coupled day–night systems remain poorly understood, particularly in environmentally sensitive alpine ecosystems. Here, we conducted a field manipulation experiment in an alpine meadow on the Tibetan Plateau, comparing illuminated and unlit conditions, and constructed temporally explicit pollination networks by integrating diurnal and nocturnal interactions. Despite no detectable changes in plant community composition, ALAN significantly restructured pollinator‐mediated interactions. At night, visitation by Lepidoptera (primarily moths), the dominant pollinators, declined sharply (−57.5%), coinciding with an overall reduction in visitation frequency (−35.4%) and a substantial decrease in network nestedness (−64.7%). In contrast, Diptera visitation increased markedly (+179.6%), partially offsetting the reduction in total visitation associated with the decline in Lepidoptera visitation. ALAN also increased nocturnal network modularity by 12.8%, indicating greater compartmentalization of nocturnal plant–pollinator interactions. Notably, these effects extended beyond the nocturnal period. During the day, total visitation remained unchanged, but strong shifts in pollinator composition emerged: Hymenoptera, Lepidoptera, and Coleoptera declined (−20.0%, −51.8%, and −61.7%, respectively), whereas Diptera increased (+46.3%). These changes were accompanied by a significant increase in network connectance (+16.1%), whereas nestedness and modularity remained unaffected by ALAN. The decline in nocturnal nestedness was primarily driven by reductions in Lepidoptera, whereas the increase in diurnal connectance was associated with increased Diptera visitation and reduced Hymenoptera visitation. Together, our results demonstrate that ALAN reorganizes plant–pollinator networks through differential responses among pollinator groups, with some groups declining while others increase in visitation and cross‐diel cascading effects, thereby altering the organization of species interactions across both nocturnal and diurnal pollination systems. Our findings highlight the importance of integrating diel dynamics into ecological network research and underscore the need to mitigate light pollution in vulnerable alpine ecosystems.

## Graphical abstract

Artificial light at night (ALAN) affects plant–pollinator interactions through taxon‐specific changes in pollinator visitation. Reduced Lepidoptera visitation restructures nocturnal pollination networks by decreasing nestedness and increasing modularity, whereas shifts in daytime pollinator assemblages increase connectance in diurnal pollination networks. Together, these results reveal cross‐diel cascading effects of ALAN on coupled day–night pollination systems.

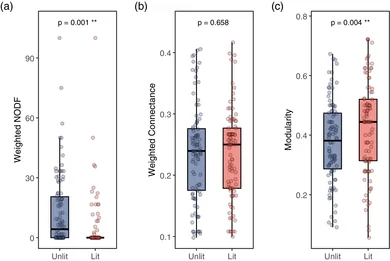

## 1 Introduction

Pollination is a key ecological process that sustains terrestrial ecosystem functions and plant diversity (Ollerton et al. 2011; Garibaldi et al. 2013). Most angiosperms globally depend on animal pollination (Ollerton et al. 2011; Tong et al. 2023), yet insect pollinators are increasingly threatened by human activities such as deforestation, agricultural intensification, and urbanization (Wagner et al. 2021). Among these threats, artificial light at night (ALAN) has emerged as a growing driver of insect decline (Grubisic et al. 2018; Owens et al. 2020; Boyes et al. 2021). By disrupting natural light–dark cycles, ALAN may alter insect biological rhythms, movement, foraging, and interspecific interactions, thereby threatening biodiversity and ecosystem processes (Gaston et al. 2015; Hölker et al. 2010; Davies and Smyth 2018; Sanders et al. 2021; Jägerbrand and Spoelstra 2023).

The effects of ALAN on pollinators are often species‐specific and can potentially alter the structure and stability of pollination networks (Sanders and Gaston 2018; Sanders et al. 2023). Immediate effects of ALAN on pollinators can contribute to network reorganization through the responses of light‐sensitive species, including changes in movement and activity that can affect community composition and species interactions (Sanders et al. 2023). For example, nocturnal moths near lights may be attracted upward and fail to visit flowers; those flying from darkness into illuminated areas can become inactive for the rest of the night (Frank et al. 2006). ALAN can also delay or prevent the natural onset of foraging and migration in moths by masking the day–night transition and effectively extending day length (Boyes et al. 2021; Meah et al. 2026). Conversely, extended day length may increase activity in diurnal insects (e.g., flies and bees). Such taxon‐specific effects reshape pollinator community structure and consequently affect network stability. Indeed, ALAN can reduce pollination network nestedness by up to 67% (Knop et al. 2017). Network‐level metrics—such as nestedness—offer valuable insights into function of plant–pollinator communities (Bascompte and Jordano 2007; Kaiser‐Bunbury et al. 2017).

Because of functional complementarity, nocturnal and diurnal pollinators can be linked through plant‐mediated indirect interactions (Knop et al. 2017). Light can influence floral traits (e.g., flowering time, scent emission; Bennie et al. 2016a, 2016b; Bloch et al. 2017), potentially altering the timing of floral resource availability and thereby affecting diurnal plant–pollinator interactions (Grognuz et al. 2026). ALAN can directly and indirectly affect herbivore populations (Bennie, Davies, Cruse, Bell, and Gaston 2018; Bennie, Davies, Cruse, Inger, and Gaston 2018; Grenis and Murphy 2019), altering plant attractiveness to pollinators and having cascading effects on flower visitation and pollination success (Rusman et al. 2018; Barber et al. 2012). By disrupting circadian rhythms or affecting nocturnal larval stages, light pollution may also negatively impact diurnal insects (e.g., butterflies, which are active as adults during the day) (Boyes et al. 2021). Thus, ALAN has the potential to indirectly affect diurnal pollinators and influence diurnal pollination networks. By merging day‐ and nighttime plant–pollinator interaction networks, Knop et al. (2017) showed that diurnal and nocturnal pollinator communities are indeed interconnected. Giavi et al. (2021) further demonstrated that ALAN can alter diurnal plant–pollinator interactions, although the direction of change depends on the plant species. Nevertheless, the consequences of ALAN for the structure and function of diurnal pollination networks remain unclear.

Alpine meadows (elevations > 3000 m) are characterized by low temperatures and short growing seasons. Plant communities in these meadows often experience pollen limitation and rely heavily on a few generalist pollinators (e.g., bumblebees) to complete pollination within a constrained time window (Körner 2003). This combination of low diversity, high dependence, and proximity to environmental tolerance limits makes alpine ecosystems particularly sensitive to ALAN disturbance. Although light pollution levels on the Tibetan Plateau are the lowest in China, this region has experienced the fastest growth in light pollution (Jiang et al. 2017), especially in its eastern part (Wang et al. 2022). This provides a unique opportunity to assess the ecological impacts of ALAN in a relatively pristine ecosystem, free from the additional anthropogenic stressors typical of urbanized landscapes. Yet, how ALAN affects the species composition and structure of both diurnal and nocturnal pollination networks in alpine meadows remains systematically unexplored.

This study employs a controlled experiment to investigate the effects of ALAN on plant–pollinator interactions and network structure in alpine meadows, focusing on three key metrics: connectance, nestedness, and modularity. Connectance reflects the density of interactions; higher connectance often implies more potential interaction pathways, contributing to system redundancy and resilience (Vázquez et al. 2009). Nestedness describes a network topology in which species with fewer links interact with subsets of the partners of more connected species (Blüthgen and Staab 2024). As a classic measure of network architecture, nestedness has been widely used to detect disturbance effects on interaction patterns, although its relationship with stability varies across ecological contexts (Blüthgen and Staab 2024). Modularity captures the degree to which a network is divided into subgroups with dense internal connections but sparse connections between modules; such a structure limits the spread of disturbances and potentially enhances resistance to perturbations (Olesen et al. 2007; Gilarranz et al. 2017; Grilli et al. 2016). Together, these structural characteristics determine how pollination networks respond to environmental change and maintain stability across different ecological contexts (Bascompte and Jordano 2007; Thébault and Fontaine 2010). We test the following hypotheses: ALAN will (1) reduce nocturnal pollinator visitation, particularly that of moths, thereby altering the structure of nocturnal pollination networks; and (2) also induce carry‐over effects on diurnal pollinator communities by shifting the relative contributions of different pollinator groups, consequently altering the structure of diurnal pollination networks.

## 2 Study Site and Methods

### 2.1 Study Site

The study was conducted in the Gannan Yellow River Important Water Supply Ecological Function Area on the eastern edge of the Qinghai–Tibetan Plateau, where the extent of light pollution has expanded significantly over the past three decades (Wang et al. 2022). The experiment was carried out at the Gansu Gannan Grassland Ecosystem National Observation and Research Station (33°40′ N, 101°52′ E; ca. 3540 m a.s.l.). The site has a mean annual temperature of 1.2°C, with mean temperatures of −10.7°C in January and 11.7°C in July; the maximum growing season temperature ranges from 23.6°C to 28.9°C. Mean annual precipitation is 620–780 mm, with the rainy season mainly from July to September. Average annual sunshine exceeds 20:00 h, and the annual frost period lasts no less than 270 days. The terrain is open and windy, with a cold climate. The vegetation is typical alpine meadow, dominated by Gramineae (e.g., *Poa* and *Elymus* spp.), Compositae (e.g., *Saussurea*), Ranunculaceae (e.g., *Anemone*), and Cyperaceae (e.g., *Kobresia*), accompanied by other forbs such as *Oxytropis ochrocephala*, *Ranunculus tanguticus*, and *Potentilla fragarioides*.

### 2.2 Experimental Design

In June 2024, we established a paired experimental design to compare lit versus unlit conditions in the research station. The ambient light radiance was approximately 0.75 × 10^−9^ W·sr^−1^·cm^−2^, indicating a low background light pollution level (data source: http://www.lightpollutionmap.info). The design included five replicates, each consisting of a treatment‐control pair spaced > 30 m apart. Each treatment‐control pair was defined as a block, with the lit and unlit plots within a block experiencing similar local environmental conditions. Thus, the five treatment‐control pairs constituted five spatial blocks that served as independent experimental replicates throughout the study. Each treatment group was centered around a commercially available 8000‐K white LED street lamp (Superfire, type: TG1‐B, nominal LED flux: 1000 lm) mounted at a height of 3 m. Identical lamp poles were installed in both lit and unlit plots, but the lamps were switched on only in the lit plots, whereas they remained switched off in the unlit plots throughout the experiment. A 5 m × 5 m area directly beneath each lamp was designated as the lit plot. This resulted in a mean ± SEM = 4.52 ± 0.23 lx (range from 0.69 to 8.99 lx) within a lit plot around the streetlamp, consistent with local urban street lighting environments (4.50 ± 0.75 lx). Corresponding control (unlit) plots of the same dimensions were established in adjacent areas unaffected by the light sources, where nocturnal illuminance measured < 0.1 lx. Illuminance levels within each lit plot were quantified using an LED light intensity meter (Model: MT‐4617LED; Prokit's Industries Co. Ltd.), with values reported in lux (lx) to align with standard lighting metrics. Lights were programmed to operate nightly from 19:30 to 05:30, with timing dynamically adjusted according to local sunset (local daylength is about 14 h). The initiation of local urban street lights was also based on the sunset. The experiment ran from July to August 2024.

### 2.3 Plant–Pollinator Interaction Surveys

Nocturnal pollination observations were conducted within the period from 30 min after sunset until 23:00, covering the peak activity period of nocturnal insects. Lit and unlit plots were sampled simultaneously. Observers walked slowly along each 1 m strip on both sides of the transect and used a high‐definition video camera to record insects landing on flowers (contacting stamens or pistils), while also recording the plant species being visited. Each plot was observed in five 20‐min sessions per sampling occasion, resulting in 100 min of standardized observation effort per plot (plots were sampled twice per month, for a total of four sampling events). Night observations were performed using night‐vision goggles and an LED headlamp alternately. Diurnal pollination observations were conducted on the same days as nocturnal observations, within the period from 13:00 to 17:00, covering the peak activity period of diurnal insects. Each plot was likewise observed in five 20‐min sessions per sampling occasion, resulting in the same 100 min of standardized observation effort per plot. The observation methods were identical to those used at night. When insect visitors touched the sexual parts of flowers, we took high‐resolution photographs using Sony HDR cameras. Pollinators were identified using an established reference collection (Bi et al. 2024; Gao et al. 2021). Voucher specimens of unfamiliar taxa (mainly nocturnal insects) were collected and later identified by entomologists to the lowest possible taxonomic level. Where species‐level identification was not feasible, individuals were assigned to morphospecies.

### 2.4 Plant Survey

Because the selected area consisted of 5 m × 5 m quadrats with relatively low plant heterogeneity, species composition and abundance varied little among sampling events. During each sampling event, we directly recorded the species richness of flowering plants (i.e., number of flowering species) and counted the flower abundance (number of flowers or inflorescences) for each flowering species within each quadrat. The list of flowering plant species in the study area is provided in Table S5.

### 2.5 Data Analysis

All analyses were conducted in R 4.3.1 (R Core Team 2024). Linear mixed‐effects models (LMMs) were fitted using lme4 (version 1.1‐35.3; Bates et al. 2015), with significance tested using lmerTest and post hoc comparisons with emmeans (version 1.10.6; Lenth 2024). Residuals were inspected for normality and homogeneity of variance, and log‐transformations were applied where necessary. Because diurnal and nocturnal communities differ substantially in species composition and ecological interactions, we analyzed these datasets separately to determine the effects of ALAN within each period.

Flowering plant communities were characterized by coverage‐standardized species richness and Shannon diversity. Diversity estimates were interpolated/extrapolated to a common level of sample coverage using the coverage‐based rarefaction and extrapolation framework implemented in iNEXT (Chao et al. 2020) before statistical analyses. These standardized diversity metrics were used as response variables in the models. Linear mixed‐effects models (LMMs) included light treatment (lit vs. unlit) as a fixed effect and block as a random intercept, and Tukey‐adjusted post hoc comparisons were performed. Separate analyses for diurnal and nocturnal datasets were conducted to assess whether artificial light differentially affected plant community structure (Magurran 2004; Morris et al. 2014; Wu et al. 2020).

Pollinator visitation was quantified as the number of flower‐visiting individuals recorded during each 20‐min survey (Gao et al. 2021), including total visitation and five taxonomic categories (Diptera, Hymenoptera, Lepidoptera, Coleoptera, and Others). Treatment effects on community‐level visitation were analyzed using linear mixed‐effects models (LMMs), with treatment as a fixed effect and block as a random intercept. Estimated marginal means (EMMs) were used to calculate treatment contrasts (lit − unlit), and 95% confidence intervals were obtained from the fitted models. Species‐specific responses were evaluated for 11 common flowering species using LMMs with plant species, treatment, and their interaction as fixed effects and block as a random intercept. Visitation frequencies were log‐transformed [log(*x* + 1)] prior to analysis. Treatment effects for each species were estimated using EMM contrasts, and effect estimates with 95% confidence intervals were visualized as forest plots. Significant positive and negative treatment effects were indicated by contrasting colors, while non‐significant effects were shown in gray. Separate analyses were conducted for diurnal and nocturnal pollination communities.

Plant–pollinator interaction matrices were constructed at the species level, with plant species represented as rows and pollinator species represented as columns. Plant–pollinator networks were constructed for each combination of time, treatment, and block using the bipartite package (v2.24; Dormann et al. 2009). Network‐level metrics included weighted nestedness (Weighted NODF; Almeida‐Neto et al. 2008), weighted connectance, and modularity. Network modularity was calculated using the quantitative bipartite modularity metric implemented in the bipartite package, using the default Beckett (2016) algorithm for modularity optimization (Dormann and Strauss 2014; Beckett 2016). To account for variation in network size and sampling effort among networks, linear mixed‐effects models (LMMs) included network size and sampling completeness as covariates. Network size was defined as the total number of taxa represented in each interaction matrix, calculated as the sum of plant species richness and pollinator species richness, because network metrics can be strongly affected by differences in network size (Blüthgen et al. 2008; Dormann et al. 2009). Sampling completeness was used as a proxy for sampling effort and was estimated using a Chao1‐based approach as the ratio of observed to estimated interaction richness (Chao 1984), thereby controlling for variation in sampling intensity among networks. Network metrics were analyzed on their original scales rather than being transformed to *Z*‐scores because our objective was to quantify the effects of ALAN in biologically interpretable units. Potential biases associated with differences in network size and sampling effort were instead accounted for explicitly through the inclusion of network size and sampling completeness as covariates. Across all networks, sampling completeness was generally high, averaging 0.48 ± 0.21 SD. Mean sampling completeness was 0.41 ± 0.20 SD and 0.41 ± 0.21 SD for diurnal lit and unlit networks, respectively, and 0.50 ± 0.20 SD and 0.58 ± 0.21 SD for nocturnal lit and unlit networks, respectively. Detailed values for network size and sampling completeness for each network are provided in the Data S1 (Date.xlsx, sheet “Network_Size_Completeness”).

Relationships between network metrics and visitation frequency (total visitation and major pollinator groups, including Diptera, Lepidoptera, Coleoptera, and Hymenoptera) were examined separately for Diurnal and Nocturnal networks. Linear mixed‐effects models were used to evaluate these relationships, with visitation frequency as the focal predictor, network size and sampling completeness included as covariates to account for variation in network dimensions and sampling effort, and block included as a random effect. Regression lines were plotted only when slopes were significant (*p* < 0.05), with *R* ^2^ and *p*‐values annotated appropriately. Networks were visualized using plotweb (Dormann et al. 2009), arranging pollinators as Diptera, Hymenoptera, Lepidoptera, Coleoptera, and Others (yellow, light blue, pink, light green, gray) and plants in a fixed order, with nodes colored dark gray for unlit and light gray for lit. Diurnal and Nocturnal networks were plotted separately, with unlit above lit, to illustrate the effects of artificial light across temporal scales.

## 3 Results

### 3.1 Impact of Artificial Light at Night on Nocturnal Pollination Networks

A total of 29 night‐blooming plant species were recorded, with no significant difference in plant species diversity between treatments (Figure S1, Table S1). Among the 2160 recorded nocturnal plant–pollinator interactions, Lepidoptera (mainly moths) dominated (1195 interactions), followed by Coleoptera (269) and Diptera (205); Hymenoptera had the lowest frequency (51). Compared to the unlit control, artificial light at night (lit treatment) significantly reduced overall flower visitation frequency by 35.4% (estimate = −4.65, *p* < 0.001; Figure 1a, Table S2). This change was primarily driven by a marked decrease (by 57.5%) in Lepidopteran visitation (estimate = −4.82, *p* < 0.001; Figure 1a, Table S2). At the species level, the reduction in Lepidopteran visitation was mainly attributed to three plant species: *Carum carvi* and *Anaphalis hancockii* (Figure 1b). In contrast, ALAN significantly increased visitation frequency of Diptera by 179.6% (estimate = 0.97, *p* < 0.001) (Figure 1a, Table S2), largely due to increased visits to *Carum carvi* and *Bupleurum chinense* (Figure 1b). ALAN did not significantly affect visitation frequency of Coleoptera, Hymenoptera, or other insect groups (Figure 1a, Table S2). When nocturnal flower visitors were classified according to diel activity patterns, the decline in Lepidoptera was largely attributable to nocturnal moths, whereas diurnal butterflies were rarely recorded at night and showed no significant treatment response (Table S3). Within Diptera, the observed increase was primarily driven by typically diurnal taxa, particularly Tachinidae, Sarcophagidae, and Syrphidae, which extended their activity into illuminated nighttime conditions (Table S3).

**FIGURE 1:**
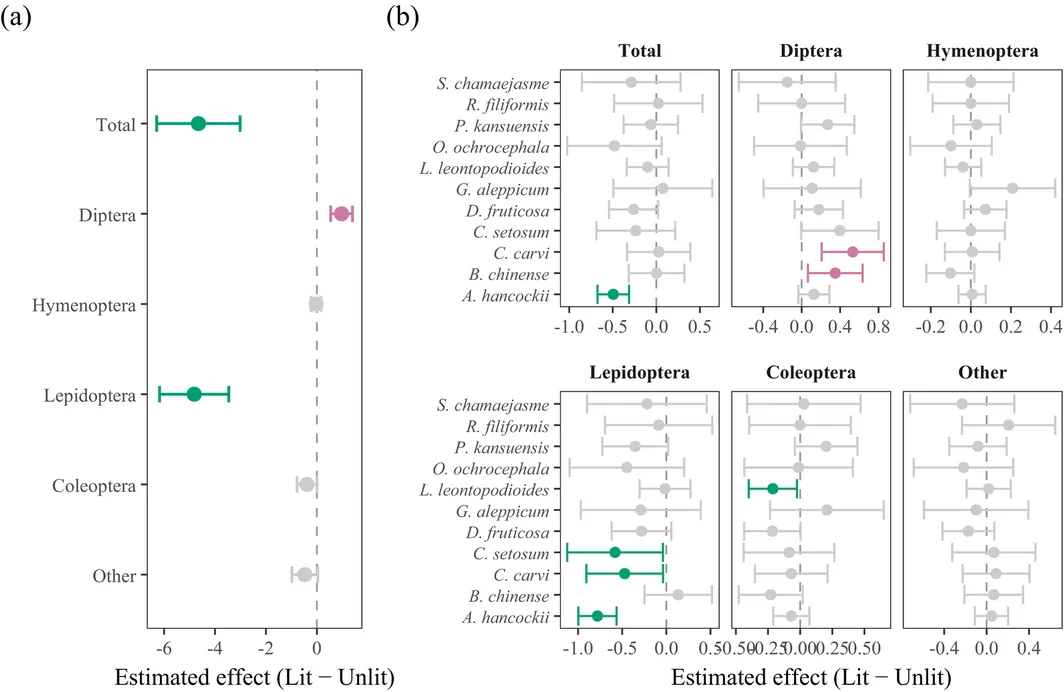
Effects of artificial light treatment on nocturnal insect visitation frequency and plant species' flower visitation behavior. (a) Effect sizes of the light treatment on total nocturnal flower visitation frequency and visitation by different insect orders (Diptera, Hymenoptera, Lepidoptera, Coleoptera, and other groups). (b) Species‐specific effect sizes of the light treatment on total flower visitation frequency and visitation by different insect orders for 11 alpine meadow plant species, arranged by total flower visitation frequency from highest to lowest (from *Stellera chamaejasme* to *Anaphalis hancockii*). Colors represent the direction of the effect: Purple indicates a significant positive effect, green indicates a significant negative effect, and gray indicates non‐significant effects. Error bars represent the 95% confidence interval, and the vertical dashed line indicates an effect size of zero. Full Latin names corresponding to abbreviated species labels (in the order shown in the figure): *Stellera chamaejasme* (*S. chamaejasme*), *Ranunculus filiformis* ( *R. filiformis* ), *Pedicularis kansuensis* ( *P. kansuensis* ), *Oxytropis ochrocephala* ( *O. ochrocephala* ), *Leontopodium leontopodioides* (*L. leontopodioides*), *Geum aleppicum* ( *G. aleppicum* ), *Dasiphora fruticosa* ( *D. fruticosa* ), *Cirsium setosum* ( *C. setosum* ), *Carum carvi* ( *C. carvi* ), *Bupleurum chinense* (*B. chinense*), *Anaphalis hancockii* ( *A. hancockii* ).

Artificial light at night significantly reduced the nestedness of the nocturnal pollination network by 64.7% (estimate = −7.64, *p* = 0.0012) (Figure 2a, Table S4) and significantly increased network modularity by 12.8% (estimate = 0.055, *p* = 0.0041) (Figure 2c, Table S4), but had no significant effects on connectance (−0.4%, estimate = −0.0004, *p* = 0.658; Figure 2b, Table S4; Figure S3). This reduction in nestedness is likely related to changes in pollinator visitation, as weighted nestedness was significantly positively correlated with both total visitation frequency (*β* = 1.07, *p* = 0.014; Figure 3a) and Lepidopteran visitation frequency (*β* = 1.29, *p* = 0.008; Figure 3c) in the lit treatment. The increase in modularity was also associated with changes in pollinator activity, as modularity was negatively correlated with total visitation frequency (*β* = −0.020, *p* < 0.001; Figure 4a), Dipteran visitation frequency (*β* = −0.023, *p* = 0.029; Figure 4b), and Lepidopteran visitation frequency (*β* = −0.019, *p* < 0.001; Figure 4c) under ALAN.

**FIGURE 2:**
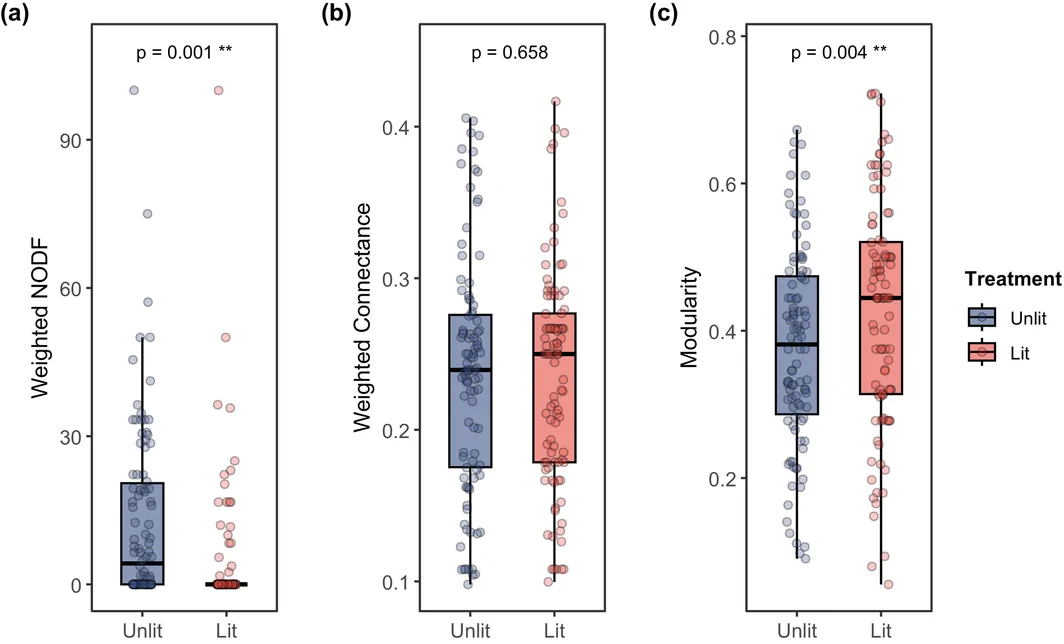
Effects of artificial light treatment on the structural metrics of nocturnal pollination networks. (a) Weighted nestedness (weighted NODF); (b) Weighted connectance; (c) Network modularity. Each panel presents boxplots of the metric under unlit (control, blue) and lit (treatment, red) conditions. Boxes denote the interquartile range, the horizontal line inside each box marks the median, and overlaid points correspond to individual replicate samples. The *p*‐value of the treatment effect is provided in each panel. Significance level: **p < 0.01.

**FIGURE 3:**
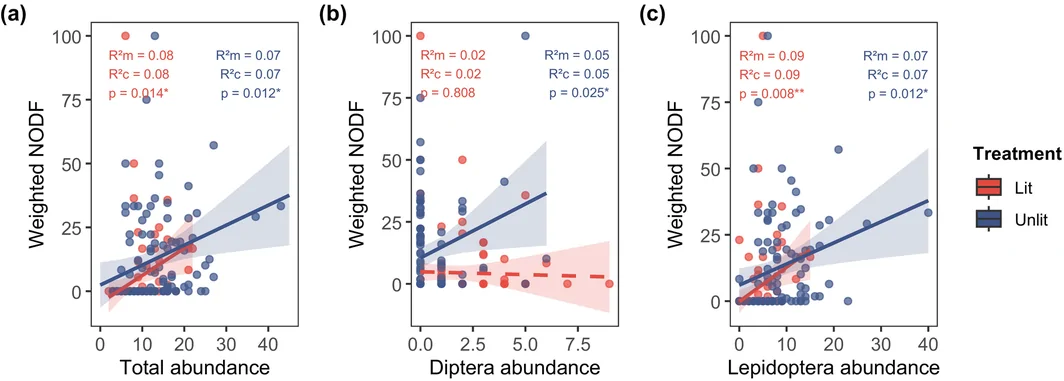
Relationships between weighted nestedness (weighted NODF) of nocturnal pollination networks and pollinator visitation frequency under artificial light treatments. (a) Relationship between total visitation frequency and weighted nestedness; (b) relationship between Diptera visitation frequency and weighted nestedness; (c) relationship between Lepidoptera visitation frequency and weighted nestedness. Red points and fitted lines represent the lit treatment, whereas blue points and fitted lines represent the unlit control. Solid lines indicate fitted regression models, and shaded areas represent 95% confidence intervals. Marginal (*R* ^2^m) and conditional (*R* ^2^c) coefficients of determination and associated *p*‐values are shown for each model. Significance levels: *p < 0.05, **p < 0.01, ***p < 0.001.

**FIGURE 4:**
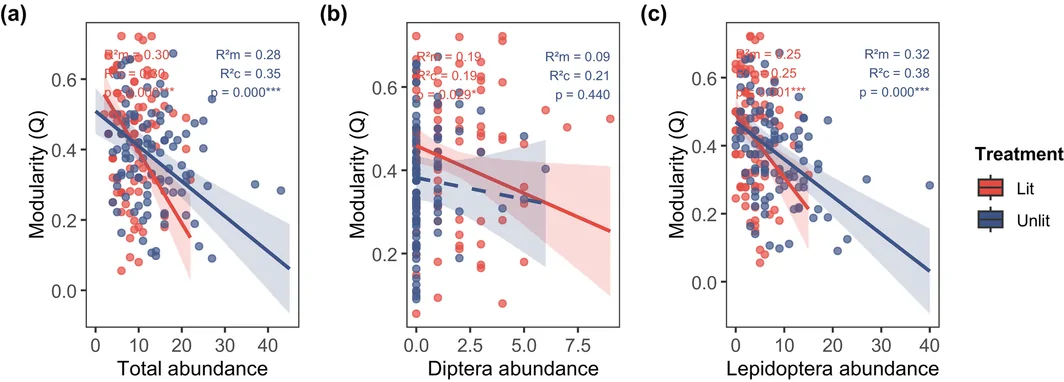
Relationships between modularity of nocturnal pollination networks and pollinator visitation frequency under artificial light treatments. (a) Relationship between total visitation frequency and network modularity; (b) relationship between Diptera visitation frequency and network modularity; (c) relationship between Lepidoptera visitation frequency and network modularity. Red points and fitted lines represent the lit treatment, whereas blue points and fitted lines represent the unlit control. Solid lines indicate fitted regression models, and shaded areas represent 95% confidence intervals. Marginal (*R* ^2^m) and conditional (*R* ^2^c) coefficients of determination and associated *p*‐values are shown for each model. Significance levels: *p < 0.05, **p < 0.01, ***p < 0.001.

### 3.2 Impact of Artificial Light at Night on Diurnal Pollination Networks

A total of 40 day‐blooming plant species were recorded, with no significant difference in plant species diversity between treatments (Figure S2, Table S1). Among the 3381 recorded diurnal plant–pollinator interactions, Hymenoptera accounted for the highest proportion (1314 interactions), followed by Diptera (1298 interactions), while Coleoptera (231), Lepidoptera (123), and other groups (482) had lower frequencies. Although overall flower visitation frequency did not change significantly between treatments (−3.8%, estimate = −0.65, *p* = 0.519; Figure 5a, Table S2), we observed significant taxon‐specific responses to artificial light at night (ALAN). Visitation frequencies of Hymenoptera, Lepidoptera, and Coleoptera decreased significantly by 20.0% (estimate = −1.46, *p* = 0.031), 51.8% (estimate = −0.43, *p* = 0.014), and 61.7% (estimate = −1.03, *p* < 0.001) (Figure 5a, Table S2), respectively. At the species level, the reduction in Hymenoptera visitation was mainly attributable to *Oxytropis ochrocephala*; the decline in Lepidoptera visitation was most evident on *Anaphalis hancockii*; and the decrease in Coleoptera visitation was primarily observed on *Stellera chamaejasme* and *Dasiphora fruticosa* , whereas Coleoptera visitation on *Oxytropis ochrocephala* increased significantly (Figure 5b). In contrast, visitation frequency of Diptera increased significantly by 46.3% (estimate = 2.44, *p* < 0.001; Figure 5a, Table S2), mainly on *Leontopodium leontopodioides*, *Cirsium setosum* , *Bupleurum chinense*, and *Anaphalis hancockii* (Figure 5b). This increase in Diptera partially offset the declines in other groups, resulting in no net change in overall flower visitation frequency.

**FIGURE 5:**
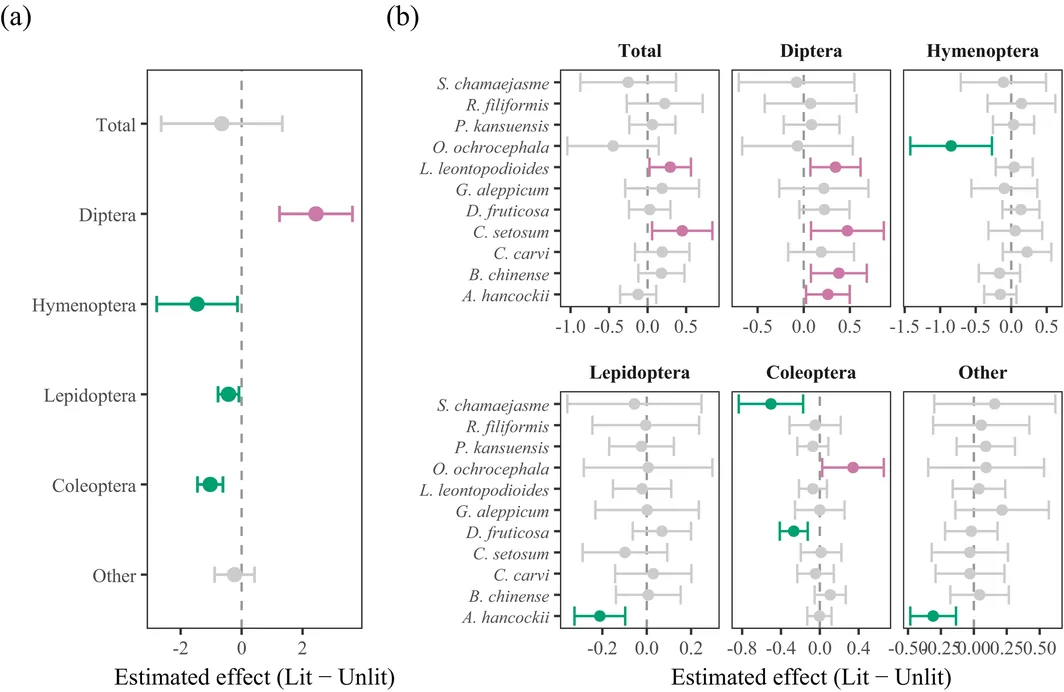
Effect sizes of artificial light treatment on diurnal insect visitation and plant species' flower visitation behavior. (a) Effect sizes of light treatment on total diurnal visitation and visitation by different insect orders (Diptera, Hymenoptera, Lepidoptera, Coleoptera, and others). (b) Species‐specific effect sizes of light treatment on total flower visitation frequency and visitation by different insect orders for 11 alpine meadow plant species, ordered by total visitation frequency from highest to lowest (from *Stellera chamaejasme* to *Anaphalis hancockii*). Colors indicate effect direction: Purple indicates a significant positive effect, green indicates a significant negative effect, and gray indicates non‐significant effects. Error bars represent the 95% confidence interval, and the vertical dashed line indicates an effect size of zero. Full Latin names corresponding to abbreviated species labels (in the order shown in the figure): *Stellera chamaejasme* (*S. chamaejasme*), *Ranunculus filiformis* ( *R. filiformis* ), *Pedicularis kansuensis* ( *P. kansuensis* ), *Oxytropis ochrocephala* ( *O. ochrocephala* ), *Leontopodium leontopodioides* (*L. leontopodioides*), *Geum aleppicum* ( *G. aleppicum* ), *Dasiphora fruticosa* ( *D. fruticosa* ), *Cirsium setosum* ( *C. setosum* ), *Carum carvi* ( *C. carvi* ), *Bupleurum chinense* (*B. chinense*), *Anaphalis hancockii* ( *A. hancockii* ).

Artificial light at night did not significantly affect modularity (−7.2%, *p* = 0.073; Figure 6c, Table S4) or network nestedness (−5.1%, *p* = 0.904; Figure 6a, Table S4), but significantly increased network connectance by 16.1% (estimate = 0.015, *p* = 0.0109; Figure 6b, Table S4; Figure S4). The increase in connectance was likely associated with shifts in pollinator composition, showing a significant positive correlation with Dipteran visitation frequency (*β* = 0.0064, *p* < 0.001; Figure 7a) and a significant negative correlation with Hymenopteran visitation frequency (*β* = −0.0047, *p* < 0.001; Figure 7b) in the lit treatment. No significant relationship was detected between connectance and Lepidopteran visitation frequency (*p* = 0.980).

**FIGURE 6:**
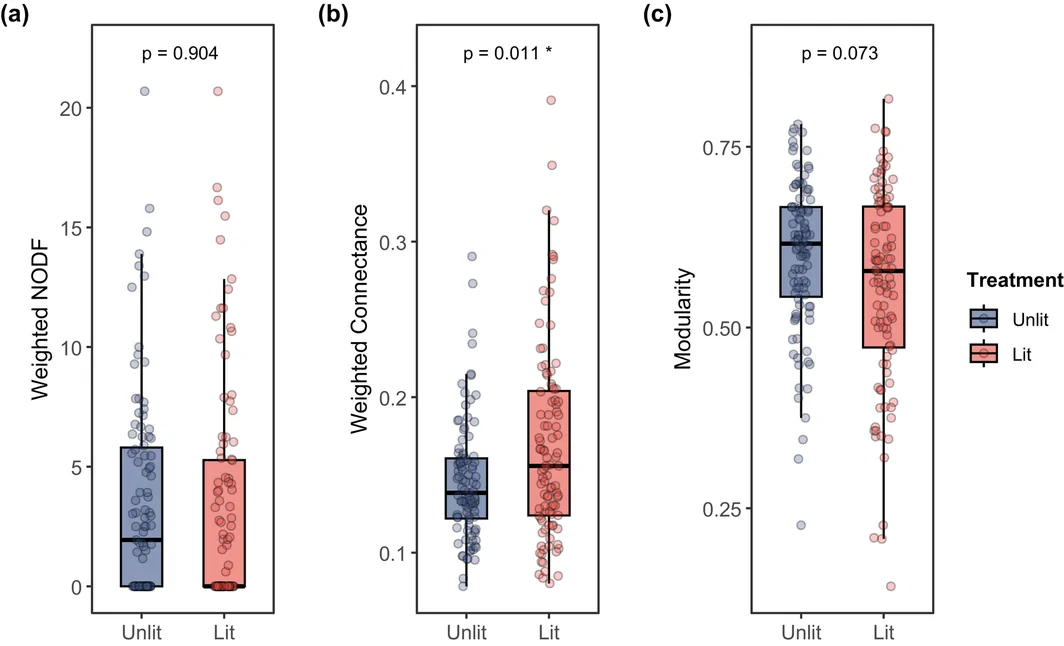
Effects of artificial light treatment on structural metrics of diurnal pollination networks. (a) Weighted nestedness (weighted NODF); (b) Weighted connectance; (c) Network modularity. Boxplots show the distribution of each metric under unlit (blue, control) and lit (red, treatment) conditions. Boxes represent the interquartile range, horizontal lines inside boxes indicate medians, and overlaid points correspond to individual replicate samples. The *p*‐value of the treatment effect is displayed in each panel; no statistically significant differences were detected between the two groups.

**FIGURE 7:**
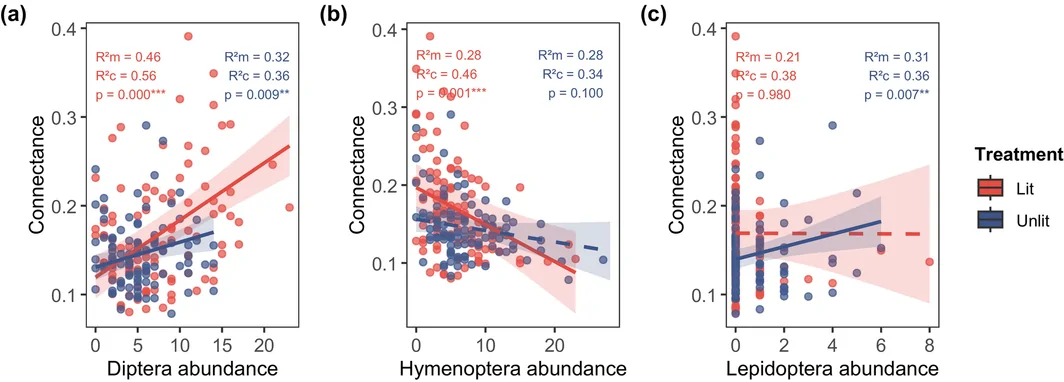
Relationships between weighted connectance of diurnal pollination networks and pollinator visitation frequency under artificial light treatments. (a) Relationship between Diptera visitation frequency and weighted connectance; (b) relationship between Hymenoptera visitation frequency and weighted connectance; (c) relationship between Lepidoptera visitation frequency and weighted connectance. Red points and fitted lines represent the lit treatment, whereas blue points and fitted lines represent the unlit control. Solid lines indicate fitted regression models, and shaded areas represent 95% confidence intervals. Marginal (*R* ^2^m) and conditional (*R* ^2^c) coefficients of determination and associated *p*‐values are shown for each model. Significance levels: *p < 0.05, **p < 0.01, ***p < 0.001.

## 4 Discussion

### 4.1 Artificial Light at Night Restructures Nocturnal Pollination Networks Through Changes in Pollinator Visitation

Our results indicate that ALAN can substantially alter the structure of nocturnal pollination networks. While the nocturnal flowering plant community remained largely unchanged, ALAN induced pronounced shifts in pollinator assemblages. Specifically, visitation by Lepidoptera (mainly moths) declined sharply (−57.5%), whereas Diptera visitation increased substantially (+179.6%), resulting in an overall reduction in visitation frequency (−35.4%). These findings indicate that the primary impact of ALAN operates through behavioral responses of pollinators, consistent with previous studies (Knop et al. 2017; MacGregor et al. 2015). The decline in Lepidoptera is likely driven by their strong phototactic behavior. Nocturnal insects rely on natural light cues for navigation, and artificial lighting disrupts orientation, leading to inefficient foraging or displacement from resource patches (Owens and Lewis 2018). In addition, increased predation risk and energetic costs under illuminated conditions may further suppress their activity. In contrast, Diptera exhibited a marked increase, likely reflecting their greater ecological flexibility and broader resource use. This opportunistic response suggests that Diptera can rapidly occupy ecological niches vacated by declining Lepidoptera under ALAN conditions.

These contrasting responses between relatively specialized Lepidoptera and more generalized Diptera translated into pronounced changes in network structure (Tylianakis et al. 2010; Poisot et al. 2016), particularly a marked decline in nestedness and a concurrent increase in modularity. Nestedness reflects the extent to which species with fewer interaction links interact with subsets of the partners of species with more links rather than maintaining exclusive interactions (Blüthgen and Staab 2024). In our study, ALAN reduced nocturnal network nestedness by 64.7%. This reduction was positively associated with both total visitation frequency and Lepidopteran visitation frequency under illuminated conditions, supporting the “abundance‐driven network structure” hypothesis, whereby interaction patterns are primarily determined by relative species abundance (Vázquez et al. 2009). The disproportionate decline in Lepidopteran visitation (−57.5%) appears to have driven the loss of nestedness, whereas the increase in Dipteran visitation was insufficient to offset this effect. Thus, ALAN triggers cascading structural changes in pollination networks by redistributing pollinator abundances and breaking down the hierarchical interaction structure typically associated with nested networks.

In parallel, ALAN significantly increased network modularity by 12.8%, indicating a more compartmentalized network structure in which interactions are concentrated within relatively isolated subgroups of species rather than distributed across the entire community (Olesen et al. 2007). The increase in modularity was negatively associated with total visitation frequency, as well as with Dipteran and Lepidopteran visitation frequency under illuminated conditions. This suggests that reduced pollinator activity weakened the inter‐module links normally maintained by highly mobile nocturnal pollinators, thereby increasing compartmentalization. Higher modularity therefore indicates a more segregated organization of plant–pollinator interactions under ALAN, which may alter the distribution of ecological processes across the network (Olesen et al. 2007; Thébault and Fontaine 2010).

Importantly, because Lepidoptera and Diptera differ in their contributions to pollen transfer, these shifts may have functional consequences beyond network structure. Reduced moth visitation could lower effective pollen transfer, potentially decreasing plant reproductive success at the community level (Knop et al. 2017; MacGregor et al. 2015; Razanajatovo et al. 2024). Consequently, ALAN may not only restructure interaction networks but also impair the ecological functions they support, with potential long‐term implications for ecosystem functioning.

### 4.2 Cross‐Temporal Effects: ALAN Propagates to Diurnal Pollination Networks

Beyond its direct effects on nocturnal interactions, ALAN also influenced daytime pollinator assemblages. Although total diurnal visitation frequency remained unchanged, strong shifts occurred among functional groups: Hymenoptera, Lepidoptera, and Coleoptera declined (−20.0%, −51.8%, and −61.7%, respectively), whereas Diptera increased (+46.3%). This indicates that ALAN induces functional reorganization rather than simple loss of activity, consistent with findings by Giavi et al. (2021), in which the effect of ALAN is not limited to nighttime interactions but can spread to diurnal plant–pollinator interactions. These cross‐temporal effects are likely mediated through multiple, non‐mutually exclusive mechanisms. First, ALAN may disrupt plant circadian regulation of floral traits, such as nectar production and scent emission (Bennie et al. 2016a, 2016b), and alter the timing of floral resource availability (Grognuz et al. 2026). Because different pollinator groups rely on these cues to varying degrees, such changes can differentially affect visitation patterns. For instance, Lepidoptera are highly dependent on floral scent and nocturnal nectar availability, making them particularly sensitive to ALAN‐induced disruptions, whereas Diptera are generally more flexible in resource use (Coux et al. 2016). Second, ALAN may directly impact diurnal insects (e.g., Lepidoptera, which are active as adults during the day) by disrupting circadian rhythms or affecting nocturnal larval stages (Boyes et al. 2021). Finally, reduced nocturnal nectar consumption due to declining moth activity may lead to a “resource surplus” during daytime, increasing nectar availability for diurnal visitors and potentially facilitating Diptera dominance (Knop et al. 2017; Owens and Lewis 2018).

These shifts in pollinator composition had clear consequences for network structure. ALAN significantly increased connectance (+16.1%) while having no significant effects on modularity or nestedness. Connectance was positively correlated with Diptera visitation and negatively correlated with Hymenoptera visitation, suggesting that increases in Dipteran activity promoted the formation of additional plant–pollinator interactions, thereby enhancing overall network connectivity. This interpretation is consistent with the known ecological role of Diptera as highly generalized pollinators capable of interacting with a broad range of plant species (Coux et al. 2016; Olesen et al. 2007). Because generalized pollinators typically interact with multiple plant species, their increased abundance can elevate the number and density of links within pollination networks, contributing to higher connectance (Bascompte et al. 2003; Olesen et al. 2007). The positive relationship between Diptera visitation and connectance observed here supports the view that generalized pollinators can enhance interaction density within ecological networks. In parallel, changes in pollinator community composition are known to influence interaction patterns and network architecture through species turnover and interaction rewiring (Bascompte and Jordano 2007; Olesen et al. 2008; CaraDonna et al. 2017, 2021; Schwarz et al. 2020). Together, these results demonstrate that ALAN can extend beyond its immediate temporal domain, restructuring diurnal pollination networks through cascading ecological effects. Our findings suggest that nocturnal artificial lighting may generate indirect effects that propagate across temporal niches, ultimately influencing daytime plant–pollinator network structure. This aligns with growing evidence that ALAN can alter ecological interactions and produce cascading effects across communities (Gaston et al. 2015; Knop et al. 2017), and highlights the importance of considering cross‐temporal linkages in understanding the full ecological impact of anthropogenic disturbances.

Nevertheless, we acknowledge that the observed network‐level changes reflect short‐term responses to ALAN exposure over a single flowering season. Whether these structural shifts persist or accumulate over longer timescales—potentially leading to enduring consequences for plant reproductive success and pollinator population dynamics—remains an open question that warrants further investigation through multi‐year experimental studies.

## 5 Conclusion

Our study provides clear evidence that artificial light at night acts as a pervasive ecological disturbance that reorganizes pollinator communities and reshapes pollination networks across both nocturnal and diurnal systems. By directly altering pollinator behavior and indirectly inducing cross‐temporal cascading effects, ALAN reduces nestedness and increases modularity in nocturnal networks, while shifting diurnal networks toward higher connectance without significantly altering nestedness or modularity. These structural changes reflect substantial reorganization of plant–pollinator interaction patterns and may carry functional consequences for pollination, given the marked differences in pollination efficiency among affected pollinator groups. Importantly, our findings highlight that the ecological consequences of light pollution extend beyond immediate behavioral disruptions to include broader community‐ and network‐level reorganization. Given the rapid global expansion of artificial lighting, mitigating its ecological impacts should be a conservation priority. Management strategies must explicitly account for light pollution as a key environmental stressor, and prioritize the development and implementation of optimized nighttime lighting regimes to reduce impacts on vulnerable ecosystems, such as alpine meadows.

## Author Contributions

**Ningna Lu:** data curation (equal), formal analysis (equal), funding acquisition (equal), investigation (equal), methodology (equal), software (equal), supervision (equal), writing – original draft (equal). **Yinyin Wang:** data curation (equal), formal analysis (equal), investigation (equal), methodology (equal), software (equal), writing – original draft (equal), writing – review and editing (equal). **Wei Cai:** data curation (equal), formal analysis (equal), investigation (equal), methodology (equal), software (equal). **Yangyang Chen:** data curation (equal), investigation (equal), methodology (equal), software (equal). **Lifang Yin:** data curation (equal), formal analysis (equal), investigation (equal), software (equal). **Xiuke Chen:** data curation (equal), formal analysis (equal), investigation (equal). **Jie Cao:** data curation (equal), investigation (equal). **Qianqian Luo:** data curation (equal), investigation (equal).

## Funding

This research was supported by the National Natural Science Foundation of China (32262270).

## Conflicts of Interest

The authors declare no conflicts of interest.

## Supporting information


**Appendix S1:** ece374368‐sup‐0001‐Appendix.docx.

**Data S1:** ece374368‐sup‐0002‐DataS1.zip.

## Data Availability

All data and R code used in this study are provided in the Supporting Information. Raw data are available in the file Data.xlsx, which includes a dedicated ReadMe sheet describing all variables, abbreviations, and measurement units used in the study. All statistical analyses and figure generation were performed using the R script R analysis‐ECE.R, which is also included in the Supporting Information.
