# Supplementary material for: Artificial Light at Night Alters Both of Nocturnal and Diurnal Pollination Networks in Alpine Meadows
Source: Ecol Evol. 2026 Oct 7;16(10):e74368. doi: 10.1002/ece3.74368 (PMC13645961; doi:10.1002/ece3.74368)
Supplement: Supplementary file 1 [file ECE3-16-e74368-s002.docx]

**Appendix**

Figure S1. Boxplots illustrating the comparison of coverage‑standardized flowering plant richness (a) and Shannon diversity (b) between Unlit and Lit treatments during the nocturnal period. Diversity metrics were standardized to a common sampling coverage using the iNEXT method (Chao et al., 2020) prior to analysis. Data points are shown as individual dots. P‑values for pairwise comparisons between treatments (Unlit vs. Lit) are displayed at the top‑right corner of each panel (LMM with Kenward–Roger df adjustment).

Figure S2. Boxplots illustrating the comparison of coverage‑standardized flowering plant richness (a) and Shannon diversity (b) between Unlit and Lit treatments during the diurnal period. Diversity metrics were standardized to a common sampling coverage using the iNEXT method (Chao et al., 2020) prior to analysis. Data points are shown as individual dots. P‑values for pairwise comparisons between treatments (Unlit vs. Lit) are displayed at the top‑right corner of each panel (LMM with Kenward–Roger df adjustment).

Figure S3. Nocturnal pollination network. The upper panel (dark gray plant boxes) represents the unlit treatment (Unlit), and the lower panel (light gray plant boxes) represents the lit treatment (Lit). The colors of the higher-level boxes (pollinators) indicate different groups: Diptera (yellow), Hymenoptera (light blue), Lepidoptera (pink), Coleoptera (light green), and Others (gray). Link colors match the corresponding pollinator box colors. Plants are arranged in a fixed order (numbered 1–43) but labels are not shown in the figure. Box width is proportional to species abundance. This order follows the plant sequence in Table S1. All analyses were conducted on species-by-species interaction matrices, which were subsequently aggregated for visualization purposes.

 Figure S4. Diurnal pollination network. The upper panel (dark gray plant boxes) represents the unlit treatment (Unlit), and the lower panel (light gray plant boxes) represents the lit treatment (Lit). The colors of the higher-level boxes (pollinators) indicate different groups: Diptera (yellow), Hymenoptera (light blue), Lepidoptera (pink), Coleoptera (light green), and Others (gray). Link colors match the corresponding pollinator box colors. Plants are arranged in a fixed order (numbered 1–43) but labels are not shown in the figure. Box width is proportional to species abundance. This order follows the plant sequence in Table S1. All analyses were conducted on species-by-species interaction matrices, which were subsequently aggregated for visualization purposes.

Table S1. Effects of nocturnal artificial lighting on plant diversity.

| Time | Response | Contrast | Estimate | SE | df | t value | p value |
| --- | --- | --- | --- | --- | --- | --- | --- |
| Diurnal | Richness_std | Unlit – Lit | -0.066 | 0.118 | 187 | -0.564 | 0.573 |
|  | Shannon_std |  | -0.058 | 0.106 | 187 | -0.550 | 0.583 |
| Nocturnal | Richness_std |  | -0.032 | 0.111 | 187 | -0.290 | 0.772 |
|  | Shannon_std |  | -0.027 | 0.100 | 187 | -0.275 | 0.784 |

Notes: Estimates represent differences between Unlit and Lit treatments (Unlit – Lit). Diversity metrics (Richness_std and Shannon_std) were standardized to a common sampling coverage using iNEXT (Chao et al., 2020) prior to analysis. All comparisons were statistically non‑significant (P > 0.05). Degrees of freedom (df) were estimated using the Kenward–Roger approximation. P‑values are from two‑sided t‑tests within linear mixed‑effects models (treatment as fixed effect, block as random effect).

Table S2. Community-level effects of artificial light at night on pollinator visitation.

| Time | Pollinator group | Estimate (Lit – Unlit) | 95% CI | P-value |
| --- | --- | --- | --- | --- |
| Diurnal | Total | –0.650 | (–2.637, 1.337) | 0.519 |
|  | Diptera | 2.440 | (1.243, 3.637) | <0.001 |
|  | Hymenoptera | –1.460 | (–2.785, –0.135) | 0.031 |
|  | Lepidoptera | –0.430 | (–0.773, –0.087) | 0.014 |
|  | Coleoptera | –1.030 | (–1.447, –0.613) | <0.001 |
|  | Other | –0.230 | (–0.884, 0.424) | 0.489 |
| Nocturnal | Total | –4.652 | (–6.291, –3.013) | <0.001 |
|  | Diptera | 0.970 | (0.544, 1.396) | <0.001 |
|  | Hymenoptera | –0.031 | (–0.223, 0.160) | 0.748 |
|  | Lepidoptera | –4.818 | (–6.176, –3.459) | <0.001 |
|  | Coleoptera | –0.390 | (–0.782, 0.001) | 0.051 |
|  | Other | –0.474 | (–0.983, 0.035) | 0.068 |

Note. Estimates are differences between Lit and Unlit treatments derived from linear mixed‑effects models with block as a random intercept; positive values indicate increased visitation under lighting. 95% confidence intervals are shown in parentheses. Significance codes: ****P*<0.001, ***P*<0.01, **P*<0.05, ns = not significant (*P*≥0.05). Community‑level abundance data were log‑transformed prior to analysis.

Table S3. Effects of artificial light at night on visitation frequency of Lepidoptera and Diptera taxa.

| Order | Class | Contrast | Estimate | SE | df | t-ratio | p-value |
| --- | --- | --- | --- | --- | --- | --- | --- |
| Lepidoptera | Moths | Unlit - Lit | –1.5665 | 0.2360 | 586.79 | –6.638 | <0.001 |
|  | Butterflies |  | –0.0128 | 0.0083 | 586.87 | –1.550 | 0.122 |
| Diptera | Mosquitoes |  | –0.0262 | 0.0311 | 583.88 | –0.842 | 0.400 |
|  | Non‑mosquito |  | 0.3083 | 0.0701 | 585.70 | 4.401 | <0.001 |

Notes: Estimates from linear mixed models with block as random intercept. Non-mosquito includes Tachinidae, Sarcophagidae, and Syrphidae.

Table S4. Fixed effects of ALAN treatment and covariates on network metrics from linear mixed-effects models.

| Time | Response | Predictor | Estimate | SE | df | t-value | P-value |
| --- | --- | --- | --- | --- | --- | --- | --- |
| Diurnal | Weighted NODF | Treatment (Lit) | -0.078 | 0.645 | 195 | -0.121 | 0.904 |
|  |  | Network_Size | 0.069 | 0.072 | 195 | 0.953 | 0.342 |
|  |  | SamplingCompleteness | 3.568 | 1.587 | 195 | 2.249 | 0.026 |
|  | Weighted Connectance | Treatment (Lit) | 0.017 | 0.006 | 191 | 2.572 | 0.011 |
|  |  | Network_Size | -0.005 | <0.001 | 195 | -6.935 | <0.001 |
|  |  | SamplingCompleteness | 0.011 | 0.016 | 194 | 0.659 | 0.511 |
|  | Modularity Q | Treatment (Lit) | -0.029 | 0.016 | 191 | -1.806 | 0.073 |
|  |  | Network_Size | 0.011 | 0.002 | 194 | 5.732 | <0.001 |
|  |  | SamplingCompleteness | -0.020 | 0.040 | 195 | -0.501 | 0.617 |
| Nocturnal | Weighted NODF | Treatment (Lit) | -7.681 | 2.344 | 192 | -3.277 | 0.001 |
|  |  | Network_Size | 0.138 | 0.439 | 192 | 0.315 | 0.753 |
|  |  | SamplingCompleteness | 7.028 | 6.259 | 192 | 1.123 | 0.263 |
|  | Weighted Connectance | Treatment (Lit) | -0.005 | 0.010 | 192 | -0.443 | 0.658 |
|  |  | Network_Size | -0.010 | 0.002 | 192 | -5.125 | <0.001 |
|  |  | SamplingCompleteness | 0.011 | 0.027 | 192 | 0.396 | 0.692 |
|  | Modularity Q | Treatment (Lit) | 0.059 | 0.020 | 189 | 2.909 | 0.004 |
|  |  | Network_Size | 0.019 | 0.004 | 191 | 4.911 | <0.001 |
|  |  | SamplingCompleteness | 0.033 | 0.054 | 192 | 0.609 | 0.543 |

Notes: All models include treatment (Unlit as reference), Network_Size, and SamplingCompleteness as fixed effects, with block as random intercept. df = Satterthwaite's degrees of freedom. Models for Diurnal NODF, Nocturnal NODF, and Nocturnal Connectance showed singular fit (block variance ≈ 0), indicating negligible block effects.

Table S5. List of plant species recorded in the study, with their family affiliations.

| No. | Species | Family |
| --- | --- | --- |
| 1 | *Anaphalis hancockii* Maxim. | Asteraceae |
| 2 | *Stellaria media* (L.) Vill. | Caryophyllaceae |
| 3 | *Stellera chamaejasme* L. | Thymelaeaceae |
| 4 | *Astragalus polycladus* Bureau & Franch. | Fabaceae |
| 5 | *Euphrasia pectinata* Ten. | Orobanchaceae |
| 6 | *Dasiphora fruticosa* (L.) Rydb. | Rosaceae |
| 7 | *Pedicularis kansuensis* Maxim. | Orobanchaceae |
| 8 | *Ranunculus nematolobus* Hand.-Mazz. | Ranunculaceae |
| 9 | *Valeriana officinalis* L. | Caprifoliaceae |
| 10 | *Geranium pylzowianum* Maxim. | Geraniaceae |
| 11 | *Potentilla potaninii* Th. Wolf | Rosaceae |
| 12 | *Carum carvi* L. | Apiaceae |
| 13 | *Taraxacum mongolicum* Hand.-Mazz. | Asteraceae |
| 14 | *Vicia sepium* L. | Fabaceae |
| 15 | *Picris japonica* Thunb. | Asteraceae |
| 16 | *Geum aleppicum* Jacq. | Rosaceae |
| 17 | *Potentilla fragarioides* L. | Rosaceae |
| 18 | *Galium verum* L. | Rubiaceae |
| 19 | *Pleurospermum camtschaticum* Hoffm. | Apiaceae |
| 20 | *Saussurea parviflora* (Poir.) DC. | Asteraceae |
| 21 | *Carpesium abrotanoides* L. | Asteraceae |
| 22 | *Erigeron acris* L. | Asteraceae |
| 23 | *Gentiana aristata* Maxim. | Gentianaceae |
| 24 | *Oxytropis ochrocephala* Bunge | Fabaceae |
| 25 | *Potentilla anserina* L. | Rosaceae |
| 26 | *Anemone rivularis* var. *flore-minore* Maxim. | Ranunculaceae |
| 27 | *Stachys japonica* Miq. | Lamiaceae |
| 28 | *Pedicularis kansuensis* f. *albiflora* Li | Orobanchaceae |
| 29 | *Bupleurum chinense* DC. | Apiaceae |
| 30 | *Cirsium setosum* (Willd.) Besser | Asteraceae |
| 31 | *Agrimonia pilosa* Ledeb. | Rosaceae |
| 32 | *Saussurea nigrescens* Maxim. | Asteraceae |
| 33 | *Saussurea amara* (L.) DC. | Asteraceae |
| 34 | *Comastoma pulmonarium* (Turcz.) Toyok. | Gentianaceae |
| 35 | *Leontopodium leontopodioides* (Willd.) Beauverd | Asteraceae |
| 36 | *Klasea centauroides* (L.) Cass. ex Kitag. | Asteraceae |
| 37 | *Halenia elliptica* D. Don | Gentianaceae |
| 38 | *Saussurea hieracioides* Hook. f. | Asteraceae |
| 39 | *Ligularia sagitta* (Maxim.) Mattf. | Asteraceae |
| 40 | *Aconitum gymnandrum* Maxim. | Ranunculaceae |
| 41 | *Delphinium kamaonense* var. *glabrescens* (W.T. Wang) W.T. Wang | Ranunculaceae |
| 42 | *Allium sikkimense* Baker | Amaryllidaceae |
| 43 | *Aster tataricus* L. f. | Asteraceae |
